# A new CAM6 + DART reanalysis with surface forcing from CAM6 to other CESM models

**DOI:** 10.1038/s41598-021-92927-0

**Published:** 2021-08-12

**Authors:** Kevin Raeder, Timothy J. Hoar, Mohamad El Gharamti, Benjamin K. Johnson, Nancy Collins, Jeffrey L. Anderson, Jeff Steward, Mick Coady

**Affiliations:** 1 National Center for Atmospheric Research, CISL/DAReS, Boulder, CO 80305 USA; 2 Spire Global Inc., 1690 38th St, Boulder, CO 80301 USA; 3 National Center for Atmospheric Research, CISL/CSG, Boulder, CO 80305 USA

**Keywords:** Climate sciences, Scientific data

## Abstract

An ensemble Kalman filter reanalysis has been archived in the Research Data Archive at the National Center for Atmospheric Research. It used a CAM6 configuration of the Community Earth System Model (CESM), several million observations per day, and the Data Assimilation Research Testbed (DART). The data saved from this global, $\sim 1^\circ$ resolution, 80 member ensemble span 2011–2019. They include ensembles of: sub-daily, real world, atmospheric forcing for use by all of the nonatmospheric models of CESM; weekly, CAM6, restart file sets; 6 hourly, prior hindcast estimates of the assimilated observations; 6 hourly, land model, plant growth variables, and 6 hourly, ensemble mean, gridded, atmospheric analyses. This data can be used for hindcast studies and data assimilation using component models of CESM; CAM6, CLM5, CICE5, POP2. MOM6, MOSART, and CISM; and non-CESM Earth system models. This large dataset (~ 120 Tb) has a unique combination of a large ensemble, high frequency, and multiyear time span, which provides opportunities for robust statistical analysis and use as a machine learning training dataset.

## Introduction

“Data assimilation” (“DA”) is the term used in many geophysical sciences for the merging of observations with a model state created by a (usually) numerical model of the physical system. The model state used in this dataset is a “hindcast” because it represents a past state^1^. The model state is referred to as “prior” to the assimilation. The result of assimilating observations into a hindcast is a “reanalysis”, which is a better description of the system than either the observations or the model state individually. Observations and model hindcasts have both valuable information and errors. Successful DA keeps the information from both and reduces the errors. It also reduces the prior uncertainty^2,3^.

The reanalyses created by the Data Assimilation Research Testbed^4,5^ (DART) use an 80 member ensemble of similar hindcasts, which leverages the power of statistics to give a more comprehensive estimate than a single hindcast can provide. The ensemble of reanalyses is a sample of the probability distribution of the physical system after the observations are assimilated (the “posterior”)^6^.

The mean of this ensemble can be viewed as the most likely weather. The spread of the ensemble tells us how much confidence to have in it (smaller spread implies more certainty) or how much variability we should expect.

**Figure 1:**
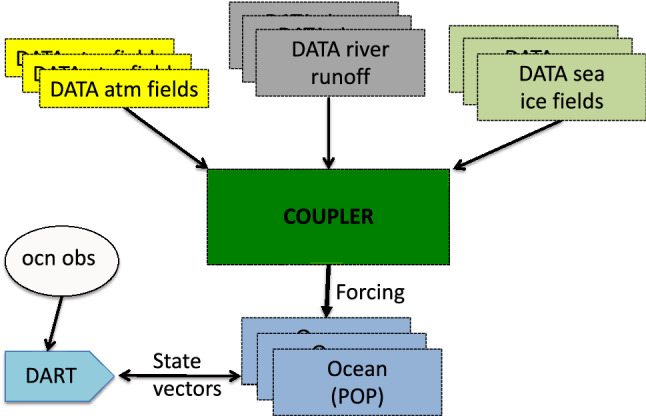
Illustration of an ensemble of data atmosphere surface fluxes from this reanalysis (and other required data) being fed to CESM’s coupler, which passes it to the ocean model. POP uses that data as an upper boundary condition during the evolution of the ocean model state. The model states are passed to DART for assimilation of ocean observations.

DA was developed to improve the initial conditions used in numerical weather forecasts^1^, but its use is spreading into studies of other Earth system components; the oceans, land, cryosphere, etc. These components are strongly forced by the atmosphere. In order to successfully model them, the atmospheric forcing must be specified correctly, both its mean and variability. The first motivation for the creation of this dataset was to provide that forcing in the context of an Earth system modeling framework, in which the atmospheric forcing can be applied to the nonatmospheric components consistently and conveniently. This is accomplished by running an atmospheric reanalysis for as long as possible and archiving all of the fluxes and other variables as frequently as required by the nonatmospheric models. Then the nonatmospheric models can be run repeatedly without the cost of regenerating the atmospheric forcing. The nonatmospheric model runs could be single or ensemble hindcasts to study the model behavior, or they could be the hindcasts used in generating reanalyses of the nonatmospheric components (see Fig. 1 for an illustration).

Several studies have found that successful ensemble DA using nonatmospheric models requires an ensemble of atmospheric forcing in order to maintain appropriate ensemble spread among the nonatmospheric model states^7–9^.

There are other useful data products created as part of the atmospheric DA process. They are archived for future repeated use. These include data which reveal the quality of the reanalyses. The quality was monitored frequently during the assimilations by comparing the 6 h model hindcasts to the observations that were about to be assimilated into those states. This comparison is made by generating ensemble model estimates of the observations *at the observation locations*, then generating statistics of the differences from the actual observations^10^. The statistics include the bias, RMSE, and “total spread”; a combination of the ensemble spread and observation error which is used to judge the size of the other statistics. These prior estimates of the observations were archived for further evaluation. They are non-gridded data, so, for convenience, the graphics generated for evaluation were also archived. Other data in this set can be used to start *atmospheric* hindcasts or data assimilation exeriments, which have many uses, including model performance evaluation, studies of atmospheric phenomena, and even exploration of a variety of assimilation algorithms which are available in DART. The last major dataset can be used to evaluate how the land model (CLM5) translates realistic atmospheric forcing into plant growth. These are described in detail in the sections that follow.

This dataset is a unique combination of a large ensemble, a multi-year time span, high frequency, and a global model constrained by observations. Such a dataset is challenging to create and archive, in terms of both computer resources and personnel time, as well as requiring careful consideration of the model definition and DA tuning. We believe that making it freely available will accelerate research in the Earth sciences.

## Methods

### Overview

#### Workflow of ensemble data assimilation

**Figure 2:**
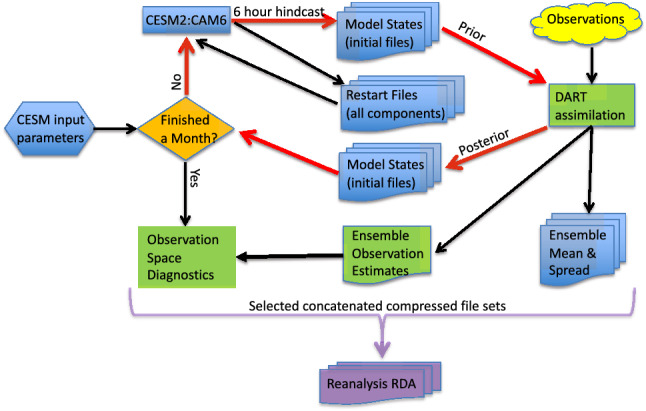
An illustration of the major parts of this ensemble data assimilation system. The red arrows highlight the core of the assimilation cycle. Black arrows indicate other data motion. “RDA” is the Research Data Archive. A cycle starts with a 6 h, ensemble hindcast, which passes an ensemble of model states to DART. DART assimilates the available observations into this prior ensemble. The resulting modified, posterior, model states are used by the hindcast model as initial conditions for the next hindcast. A flowchart of “Observation Space Diagnostics” (lower left box) is shown in Fig. 6.

This reanalysis dataset is the result of a 9 year long series of assimilation cycles. Each cycle (see Fig. 2) consists of a 6 h, ensemble hindcast calculated by a CAM6 configuration of CESM2.1 (respectively; Community Atmosphere Model v.6, Community Earth System Model v2.1^11^) (details in “Hindcast model”). The resulting model states are passed to DART, which modifies them to be more consistent with the set of observations available at that time (details in “Observations” and Table 7). At the end of each month of cycling, the results are evaluated. If they pass inspections, they are repackaged into more convenient formats and archived. The algorithms employed by DART are described in “Assimilation software”. Selected results from the hindcasts and assimilation are saved for immediate or later use, as described in the rest of the paper.

#### Context for understanding the data products

Given the major anticipated uses of these products in CESM and DART contexts, the data organization and description are motivated by the *files* created and used by those packages, instead of by the individual fields which may interest some researchers. In fact, many files contain a large number of variables which only have meaning in the context of CESM or DART, so this Data Description will not describe those. They are intended to be used, but not evaluated out of context. It *will* describe other fields which may be interesting outside of their usefulness in those packages.

The millions of files created by the assimilation process have been thinned, condensed, and packaged into more convenient units of data to facilitate archiving, discovery of desired data, downloading, and use.

Since the data spans 9 years, it includes a wide range of actual atmospheric behavior ranging from regional fronts and storms to multiyear, global patterns like El Niño. The various data products have frequencies ranging from hourly (surface forcing) to four times/day to capture the daily cycles (e.g. the ensemble mean and sets of observations) to weekly (e.g. ensembles of CESM restart files). There is enough data saved weekly that the discarded data could be recreated at reasonable expense, if needed.

The high frequency, *ensemble mean* data sample the climatological distributions, while the *ensemble* adds information about the local model attractor structure, given the observational constraints (the model attractor is the climatology generated by the model when it is free to run without being constrained by observations). This combination of frequent mean and less frequent full ensemble describes both large-scale and small-scale characteristics of states related to the model attractor when it is constrained to be close to observations. This is a CMIP6^12^ model configuration of CESM2.1^11^. There exist other samples from this model’s climatological attractor, which were *unconstrained* by atmospheric observations, other than the tuning of parameterizations to maximize the performance as a climate model. For example, see “MultiModel Large Ensemble”. This dataset can be compared to those to identify model biases and estimate the fidelity of the model in years long simulations.

#### Software environments

Almost all of the data products are in NetCDF-4 compatible format, so a wide variety of tools can be used to access and manipulate the data. However, DART and CESM are both software environments which have been developed to provide flexible and effective use of these products. They also include user support in the forms of online and embedded documentation, tutorials, and issue forums. Making use of these environments can accelerate research using these datasets.

The core programs of both packages are written in Fortran for efficiency, especially when running on supercomputers. Both packages are usually run in Unix environments (DART scripts are mostly csh). CESM scripting requires Python.

DART is designed to efficiently use MPI parallelism, but not threading (e.g. OpenMP), so CESM is configured to use the same parallelism. On NCAR’s Cheyenne supercomputer we found it highly beneficial to set two MPI environment variables to larger values than the defaults. In CESM’s env_mach_specific.xml:

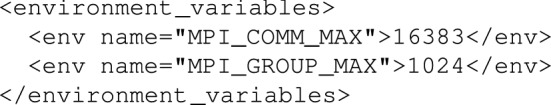



### Assimilation components description

#### Observations

The observations and retrievals (quantities derived from raw observations) used to create this dataset were gathered from a variety of publicly available sources. These include wind and temperature observations from airplanes, radiosondes, and retrievals from satellite observations, and Global Positioning System (GPS) refractivity observations (basically density) from COSMIC satellites, for example.

The sources of the observations were:

$$\triangleright $$
From NCEP’s PREPBUFR files (prepqm) in NCAR’s Research Data Archive: (ds090.0):
  - radiosondes     from balloons, mostly over land.
  - ACARS                   from commercial aircraft, mostly over North America.
  - AIRCRAFT    from commercial aircraft.
  - CDW            Cloud drift winds from GOES satellites.
$$\triangleright $$
From COSMIC: Global Positioning System satellites radio occultation. Importing them to DART is described in your_DART/observations/obs_converters/gps/gps.html
$$\triangleright $$
From AIRS: Infrared soundings from the AQUA satellite (Aqua_AIRS_Level2, AIRS2RET.006). Importing them to DART is described in your_DART/observations/obs_converters/AIRS/AIRS.html. In addition, the observations were thinned by using only every ninth observation along track and every tenth observation cross track.

**Figure 3:**
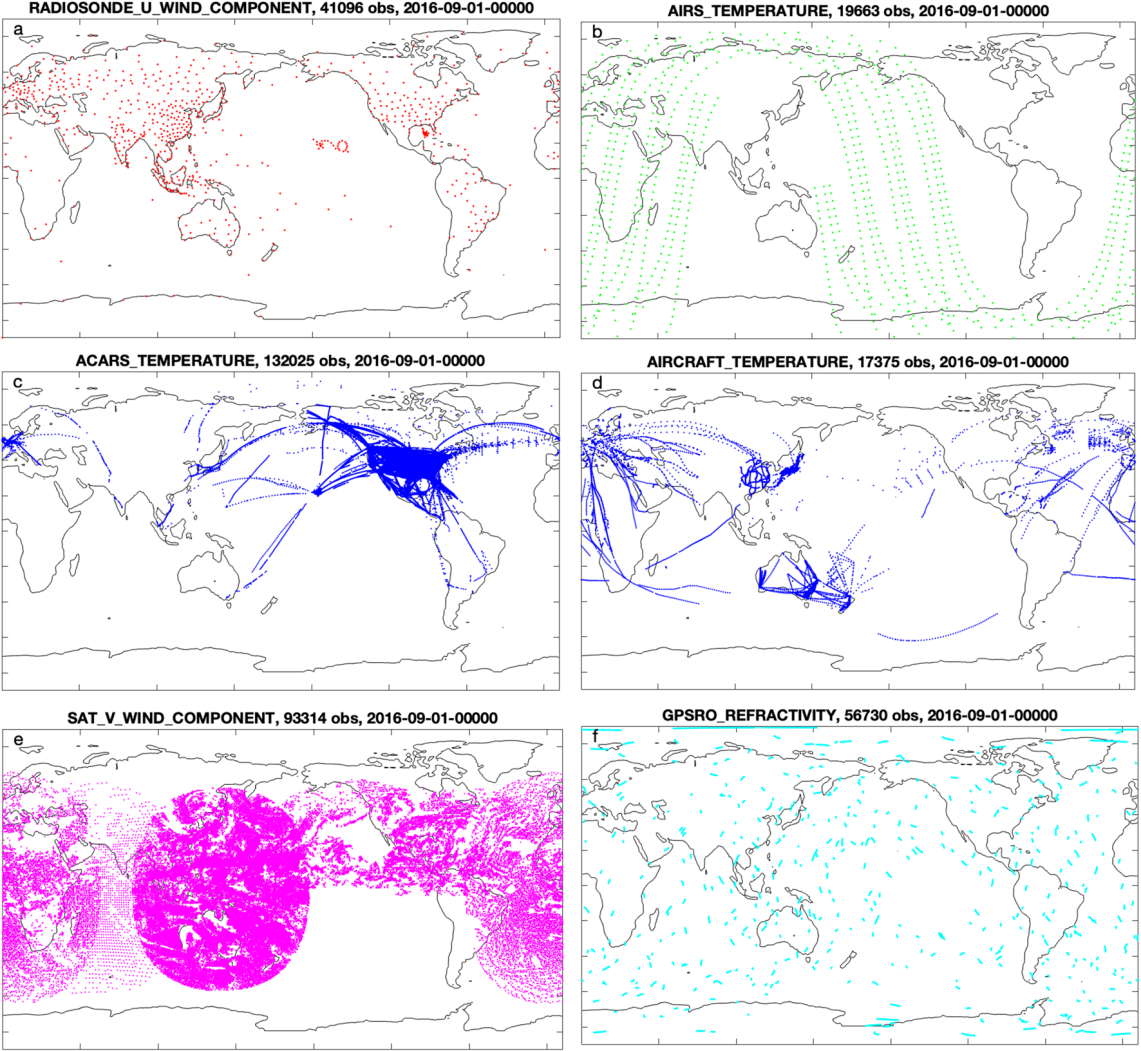
Observation distributions from a subset of the observation types from a 6 h assimilation window. The number of observations shown in each picture is printed at the top of each picture. A dot may represent a of column of observations. The continental outlines are the elevation = 0 contour of Matlab’s topo.mat data file. They were drawn by Matlab scripts in DART. See *Code availability* for version information.

The list of observation types used by this reanalysis is in Table 7. The observation error of each observation was taken from the original data source, and is stored with the observation values in this archive. See “Observation sequence files” for details. In some cases the error specified is larger than it could be. This is discussed briefly in “Observation space diagnostics”. The only harm of this is that those observations will be underweighted in the assimilation process; other similar observations and the model will have stronger influences. There are several million observations per day. A subset of the observations from one assimilation time are shown in Fig. 3.

The observations are repackaged into binary files with a format that satisfies DART’s requirements. Conversion to ASCII is available, via a DART program, and interpretation of the contents is available in the DART documentation. See “Observation sequence files”.

#### Hindcast model

The released version of CESM2.1 used here contains models of all the parts of the Earth system that influence the weather: atmosphere, ocean, land, sea and land ice, biogeochemistry, etc. Communication between these components is controlled by the “coupler”. Each component can be “active” (calculating the evolution of the model state), “data” (reading a model state that was generated elsewhere), or “stub” (no data passed to or from that component). The mode of each component is defined by a “compset” (component model set, documented here). This reanalyis used the compset HIST_CAM60_CLM50%BGC-CROP_CICE%PRES_DOCN%DOM_MOSART_SGLC_SWAV which is parsed as in Table 1 . HIST does not describe a component. It means that initial conditions are historical (not from a climate projection).

**Table 1:** The definition of the component set, which defines the hindcast model used in this reanalysis.

Component	Mode	Description
CAM60	Active	Community Atmosphere Model v.6.0.34
CLM50%BGC-CROP	Active	Community Land Model v. 5.0 in Biogeochemistry-with-crops mode
CICE%PRES	Active	Community Ice Model, in “present day” configuration. (Predicts ice thickness, but see DOCN)
DOCN%DOM	Data	Sea surface temperature and sea ice coverage data, read from files (see “Hindcast model”)
MOSART	Active	River runoff model
SGLC	Stub	Land glacier
SWAV	Stub	Water waves

CAM’s “resolution” variable defines the horizontal grid and the dynamical core, which in this case is the Finite Volume core, which is defined on a logically rectangular grid with spacing $\sim 0.9^\circ$ in latitude and $1.25^\circ$ in longitude. The land model (CLM5) uses the same grid. The vertical resolution is defined by the choice of the atmospheric component. For CAM6-FV it is 32, hybrid $\sigma$ + pressure levels, which are terrain following (“$\sigma$”) near the surface (the pressure on a level varies from place to place), constant pressure surfaces near the top, and a mixture of the two in the middle. The levels range from 7.44 hPa *above the model ground* up to a height of 3.64 hPa.

The data ocean model reads sea surface temperature (SST) and sea ice coverage data from files containing AVHRR data. The spatial resolution of this data is higher $(0.25^\circ )$ than CAM. The temporal resolution is nominally daily, but represents an ocean state which is a weighted average of data from several days. See “Sea surface temperature and ice coverage” for details.

Additional external forcing of CAM includes specified aerosols, greenhouse gases, and volcanic forcing, from CESM datasets. These are historical data for dates through 2014, and use CMIP6 scenarios after that. More information about these datasets is in “Files that force CAM”.

#### Assimilation software

DART provides an extensive choice of algorithms for ensemble Kalman filter data assimilation^4^. Based on extensive experimentation, the choices in Table 2 define the assimilation algorithm which generated these reanalyses.

**Table 2:** The assimilation parameters which have the most impact on the assimilation and the values used for this reanalysis. They are set in fortran namelists within the input.nml file.

Parameter	Value	Description
filter_kind	1	Ensemble Adjustment Kalman Filter (EAKF)^13^
ens_size	80	Number of ensemble members
num_groups	1	Number of groups into which members are divided
inf_flavor	5	Enhanced, spatially and temporally varying, adaptive, prior, covariance inflation^14^.
inf_lower_bound	0.0	Inflation mean lower bound
inf_upper_bound	100.0	Inflation mean upper bound (is never reached)
inf_sd_lower_bound	0.6	Inflation standard deviation lower bound
inf_sd_max_change	1.05	Inflation standard deviation maximum change in an assimilation cycle
inf_damping	0.9	Inflation damping (acts opposite of inflation to enhance temporal adaptiveness)^15^
horiz_dist_only	.false.	Include the vertical distance in calculations of the distance between observations and state variables
cutoff	0.15	Half width (in radians) of the spatial localization function^16^
vert_normalization_scale_height	1.5	Scaling factor relating vertical distance to horizontal in localization calculations. 1.5 scale heights = 1 radian in the horizontal
sampling_error_correction	.true.	Apply a correction to account for the limited ensemble size^17^
input_qc_threshold	3.0	Reject observations having a quality control value larger than this
outlier_threshold	3.0	Reject observations which are more than this number of total spread standard deviations different from the ensemble mean

The model state is chosen to be the set of prognostic variables in CAM, which are required to define all other variables at the beginning of a hindcast. The models’ states are stored in CAM “initial” files, from which both CAM and DART read them, and to which both write updated model states. See Table 6 for the list of variable names, which define the model state in a namelist in the input.nml file. Although the model state is defined using only atmospheric variables, and all observations are atmospheric, the land and sea ice variables evolve consistently with the atmosphere, due to the coupling in the model. Those variables are passed along from cycle to cycle by being stored in those components’ “restart” files.

Note that there is no specification of the error covariances to use in the assimilation. The reason is that in the ensemble Kalman filter the background error covariance is never explicitly calculated. It is implicit in the algorithm, which calculates, at each time and for each observation, the correlation between the ensemble of model estimates of the observation and the ensemble of each model state variable. Large correlations result in large increments added to the state variable, and small correlations result in small increments.

#### Data management

The assimilation process generates a large amount of data in the form of large numbers of “small” files (Kbytes to several Gbytes), which are useful in the moment, but only some of which need to be archived. Those which need to be archived have been combined into files which have a convenient size for future use and make the data easier to find in the archive. In addition, they are compressed for efficient storage and transfer to where they are needed. This is described in “Data records”.

### CESM model state at the start

The CESM compset used in this reanalysis has active components with a broad range of dynamical memory, due to differing levels of nonlinear behavior and inertia in the physical systems. The atmosphere has some long-lived constituents, which influence the weather, but their persistent influence is overwhelmed by the model’s perturbation error growth. Starting hindcasts with tiny differences in initial conditions and running them for 2 weeks results in model states that appear to be unrelated, other than being in the same season. There is also a relatively high density of atmospheric observations, so the model state can be brought into agreement with them in a time span measured in days. In contrast, the land model has large reservoirs of some constituents, which react slowly and non-chaotically to forcing from the atmosphere. There is a relatively low density of observations, especially below a few meters below the surface, so bringing an arbitrary land model state into agreement with observations can take months to decades of assimilation. This reanalysis dealt with those issues by starting an atmospheric assimilation 6 months before the reanalysis start date. This “spin up” assimilation used an ensemble of CLM model states, which itself had already been spun up for 12 years, forced by a “2 degree” CAM4 ensemble reanalysis.

### Validation

We include a subsection about validation in “Methods” section because the results of our validation calculations are, themselves, one of the products in this dataset. They are available for further use, so the methods used to derive them should be described here. Further validation details are in “Technical Validation”.

One of the most important aspects of the assimilation to keep in mind is that it balances the uncertainty in the model, as represented by its ensemble spread, and the uncertainty in the observations, as represented by the observation error variance. This balance is a function of field, location, and time, due to the heterogeneous distribution of observations and the range of weather phenomena which the model must describe. If the uncertainty in the model becomes too small, the assimilation will reject observations that appear to be outliers, even though they are valid observations. This can allow the spread to become even smaller, and so on, until the model has diverged from the observations and ignores almost all of them, in which case the assimilation has failed. This is prevented by the ensemble spread “inflation” algorithm^14^.

#### Observation space diagnostics

Assimilation health can be monitored by processing the assimilation output periodically to examine the fraction of observations used as a function of time, and the bias and RMSE relative to the observations. The latter are calculated by comparing the model mean estimates of the observations to the actual observations at the observation locations, i.e. in “observation space”. We do not use the posterior model state because it may be overfit to the observations, which would give an overly optimistic representation of the assimilation. The assimilation results are compared against each observation type separately, e.g. a figure may show a time series of the bias of the model estimates of temperature relative to the radiosonde temperatures that were assimilated, but not relative to the aircraft temperatures, which are taken at different locations and times and may have different biases. Details of the DART diagnostic software and how to do further validations are described in “Observation sequence files”.

**Figure 4:**
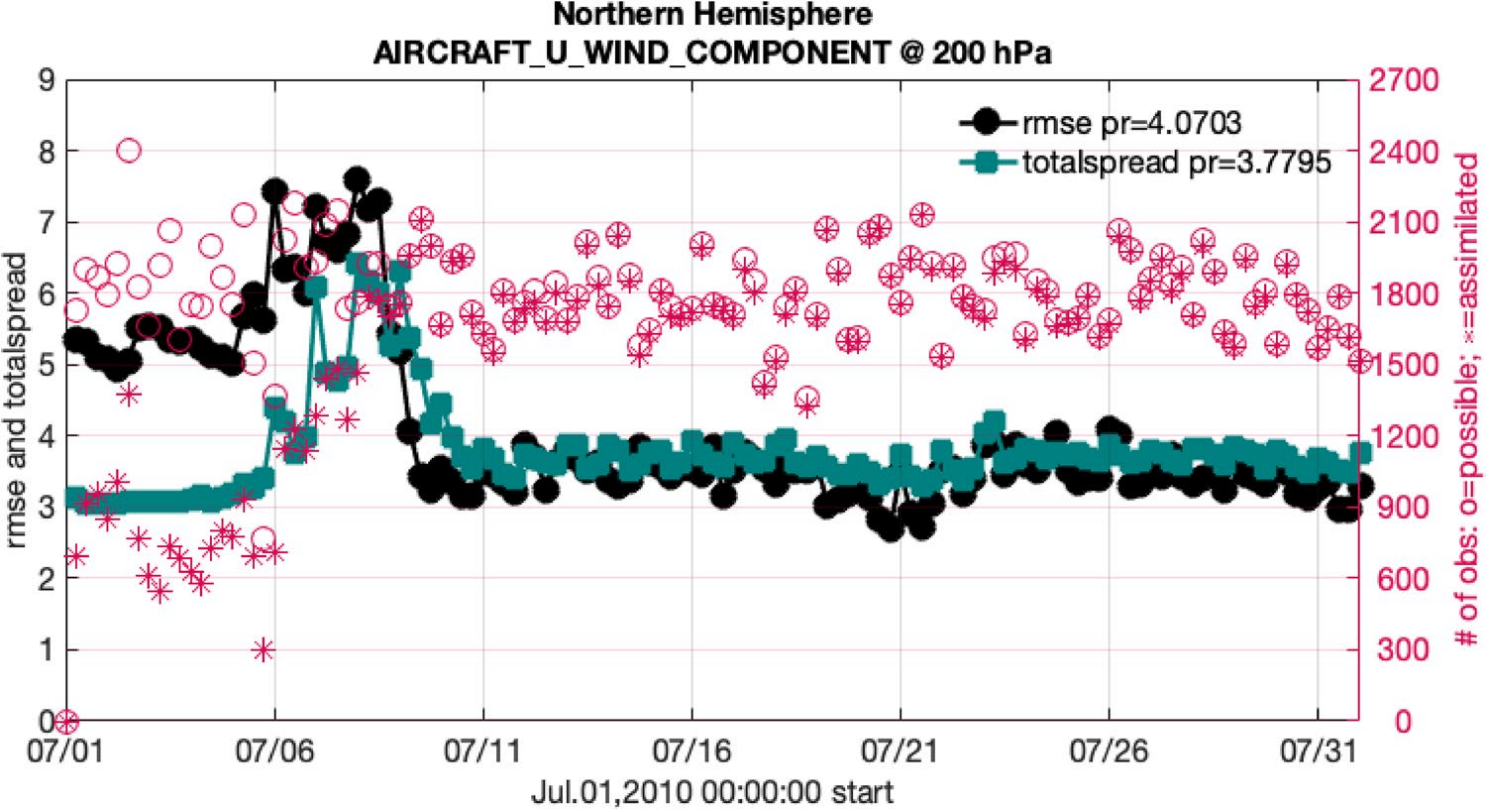
Time series of quantities, which are derived from an assimilation, and which are described in “Observation space diagnostics”. The red circles and plusses mark the number of observations available and assimilated at each time (right axis). The black line is the root mean square error of the prior model estimates of the zonal wind ($\text{ms}^{-1}$) relative to the wind observed by AIRCRAFT (left axis). The green line is the total spread, described in the same section. These quantities were derived from a subdomain of the model; north of $20^\circ$ N and from approximately 225–275 hPa high. The statistics in the header pool all of the observations in this time span, including the spinup period, so they are not representative of the statistics in the rest of the reanalysis.

These ideas are illustrated in Fig. 4, which is derived from the “spinup” assimilation, which ensured that the initial ensemble of the reanalysis represented a stable pattern, with no remnants of the arbritrary initial conditions. The figure displays a useful quantity we call “total spread”. Since both the hindcast and observations have uncertainty associated with them, it is helpful to view them as probability distributions, each with its own spread around its most likely value, those being the ensemble spread ($\sigma _{model}$) and the observation error standard deviation ($\sigma _{obs}$). Whether the two distributions are distinct or essentially indistinguishable depends on both spreads, as well as the difference between the means. The total spread is a combination of the two spreads, $\sigma _{total} = \sqrt{\sigma _{obs}^2 + \sigma _{model}^2}$, which can be used as a measure of whether the RMSE is “large” or not. The model state can be arbitrarily accurate, given enough good observations, so the RMSE can be arbitrarily small. The totalspread, however, is always at least as large as the observation error standard deviation, which is independent of the ensemble model state and the assimilation. So we expect the RMSE to be less than or equal to the total spread. In practice, the RMSE is often less than the total spread, which indicates that the observation error is larger than it needs to be.

In Fig. 4 the initial ensemble has a tiny spread, so the total spread is small. This causes the assimilation to ignore more than half of the observations because it considers them to be impossible outliers. Note that the RMSE of the initial ensemble is relatively large, even though the observations that are farthest from the ensemble have been excluded from the RMSE calculation because the observations were rejected. So the ensemble has very high confidence in a *poor* estimate of the temperature. However, almost half of the observations *are* assimilated, so the model state is pushed towards those observations. At the same time, the assimilation sees that the model estimate is incorrect and it increases the size of the ensemble spread (reduces its confidence) via the adaptive inflation algorithm. Around day 5 the ensemble spread has become large enough that it allows the number of observations assimilated to increase. Simultaneously the RMSE rises, as larger numbers of observations are used and they reinforce the conclusion that the ensemble estimate is poor. Around day 7 the totalspread has increased enough to permit the assimilation of almost all of the observations, and to allow the ensemble to agree with them. By day 10 the RMSE has fallen by about half and virtually all of the observations are being used. This signals to the assimilation that the ensemble now has a good estimate, so it allows the ensemble spread to decrease. By the end the ensemble has high confidence in a *correct* estimate, which is confirmed by the small RMSE. At that point the assimilation is considered to be healthy: almost all of the observations are being assimilated and the RMSE is approximately the same size as the total spread.

If the RMSE in that figure is replaced by the bias, then it indicates possible bias in the model and/or the observations. If the biases relative to the observations from a variety of platforms (radiosondes, satellites, ...) are of the same sign, then it is reasonable to conclude that the model is biased. If the biases are of different signs, then it seems likely that some of the observations are biased, and possibly the model too.

This dataset was checked after every month of assimilation. The graphical results of that are available as described in “Statistical representations of observation space performance”. The files which were processed (“observation sequence files”) are also available for further evaluation. There were no instances of the ensemble diverging catastrophically from the observations. In fact, the filter program never failed for algorithmic reasons (there were plenty of machine hardware and software failures).

#### State space

The assimilation can cycle through months in a fairly stable, healthy state, but occasionally there are features in the observation space diagnostics which merit investigation. For example, the number of radiosonde observations might suddenly increase, and, possibly with it, the RMSE. This is often caused by the appearance of a hurricane, which prompts weather services to take extra observations, as can be seen in Fig. 2a (in the Gulf of Mexico). This can be verified by examining the assimilation output which is on the model grid, often referred to as “state space”. If a hurricane is visible, and maps of the observation locations coincide with it, then the issue is resolved. Other features of the assimilation are more subtle, and may indicate that the quality of an observation type is evolving, or that something about the model is changing from year to year, such as the external data forcing files it reads.

#### Community evaluation

This ensemble reanalysis is a large and varied dataset, which has been evaluated for only basic signs of correctness. There are many opportunities for researchers at all stages of their careers (including students) to use or probe the data and publish the results. We welcome interactions ranging from simply learning about results, to answering questions, to in–depth collaborations.

## Data records

### Archive location and access

The reanalysis dataset has been archived in the Research Data Archive (“ds345.0”^18^) at the National Center for Atmospheric Research (NCAR). It is freely available, but a simple registration is required. The following subsections describe the data products as they are organized in the RDA site, which is consistent with the organization of the CESM short term archive. There are subdirectories for various groups of archived files. In each group directory there are subdirectories for either the ensemble members or dates archived there. A key to the outline of the archive is:

- “RDA PRODUCT name” (files_group_name [a link]),
  - ensemble member or date subdirectories
    - data files

Where, in the following,

INST: = 0001 $$\ldots $$ 0080
YYYY: = 2011 $$\ldots $$ 2019
MM: = 01 $$\ldots $$ 12
DD: = 01 $$\ldots $$ 31
SSSSS: = 00000, (occasionally 64800)

The files in the subdirectories are described in the subsections below. Follow the files_group_name links.

- “CESM Flux Coupler (cpl7) Files” (cpl)
  - cplINST
    - f.e21.FHIST_BGC.f09_025.CAM6assim.011.cpl_INST.hr2x.YYYY.nc.gz
    - f.e21.FHIST_BGC.f09_025.CAM6assim.011.cpl_INST.ha2x3h.YYYY.nc.gz
    - f.e21.FHIST_BGC.f09_025.CAM6assim.011.cpl_INST.ha2x1hi.YYYY.nc.gz
    - f.e21.FHIST_BGC.f09_025.CAM6assim.011.cpl_INST.ha2x1h.YYYY.nc.gz
    - f.e21.FHIST_BGC.f09_025.CAM6assim.011.cpl_INST.ha2x1d.YYYY.nc.gz
- “CESM Atmosphere (CAM6.0) Files” (atm)
  - atmYYYYMM
    - f.e21.FHIST_BGC.f09_025.CAM6assim.011.cam_allinst.e.forecast.YYYY-MM-DD-00000.tar
- “External System Processing (DART) Files” (esp = DART)
  - espYYYYMM
    - Diags_NTrS_YYYY-MM.tgz
    - f.e21.FHIST_BGC.f09_025.CAM6assim.011.cam_obs_seq_final.YYYY-MM.tgz
    - f.e21.FHIST_BGC.f09_025.CAM6assim.011.dart.e.cam_forecast_mean.YYYY-MM.tar
    - f.e21.FHIST_BGC.f09_025.CAM6assim.011.dart.e.cam_forecast_sd.YYYY-MM.tar
    - f.e21.FHIST_BGC.f09_025.CAM6assim.011.dart.i.cam_output_mean.YYYY-MM.tar
    - f.e21.FHIST_BGC.f09_025.CAM6assim.011.dart.i.cam_output_sd.YYYY-MM.tar
    - f.e21.FHIST_BGC.f09_025.CAM6assim.011.dart.rh.cam_forecast_priorinf_mean.YYYY-MM.tar
    - f.e21.FHIST_BGC.f09_025.CAM6assim.011.dart.rh.cam_forecast_priorinf_sd.YYYY-MM.tar
    - f.e21.FHIST_BGC.f09_025.CAM6assim.011.dart.rh.cam_output_priorinf_mean.YYYY-MM.tar
    - f.e21.FHIST_BGC.f09_025.CAM6assim.011.dart.rh.cam_output_priorinf_sd.YYYY-MM.tar
- “CESM Land Model (CLM5.0) Files” (lnd)
  - lndINST
    - f.e21.FHIST_BGC.f09_025.CAM6assim.011.clm2_INST.h0.YYYY.nc.gz
    - f.e21.FHIST_BGC.f09_025.CAM6assim.011.clm2_INST.h1.YYYY.nc.gz
- “CESM Restart Files including Initial Files” (rest)
  - restYYYYMM
    - f.e21.FHIST_BGC.f09_025.CAM6assim.011.INST.alltypes.YYYY-MM-DD-00000.tar
    - f.e21.FHIST_BGC.f09_025.CAM6assim.011.infl_log.alltypes.YYYY-MM-DD-00000.tar

### File naming conventions

In each of those directories are files, whose names roughly follow the CESM file naming convention (‘[]’ = optional part):

CASENAME.model[_INST].filetype[.subtype].DATE.extension[.compression]

Tar (tape archive) files contain files which also follow this convention. The parts of the file name can be interpreted using Table 3. An example is f.e21.FHIST_BGC.f09_025.CAM6assim.011.cam_allinst.e.forecast.2011-01-03-00000.tar. More examples are listed in the relevant subsections, below.

**Table 3:** The parts of CESM file names, their interpretation, and illustrative examples. The many filetypes will be described in the file description subsections, below.

Part	Description	Examples
CASENAME	CESM ‘CASENAME’	f.e21.FHIST_BGC.f09_025.CAM6assim.011
model	CESM component model	cpl, cam, clm, $\ldots$
INST	The [optional] ensemble member(s)	0080, allinst (‘instance’ is the CESM name for ensemble member.)
filetype	Abbreviated type of file	‘i’nitial, ‘e’xternal system processing, ha2x1d = ‘h’istory ‘a’tm ‘to any’ ‘1d’ay frequency.
subtype	Refinement of the filetype	‘forecast’ file from filetype ‘e’
DATE	Hindcast date and time label	2011-01-03-00000, 2019-12
extension	Abbreviation of the file format	nc (NetCDF), tgz (gzipped tar file)
compression	Abbreviation of the compression used	gz (gzip)

### Surface forcing files

The physical system components of CESM influence each other via fluxes and component variables at the Earth’s surface. CESM’s coupler mediates this communication and can write those fluxes and variables to “history“ files for later use. This reanalysis produced the five different coupler history files needed to run combinations of CESM components which require a data atmosphere (with a few, rarely used exceptions). These files are intended to be used only in this way, so we will not describe each variable in each file, but will focus on how to use them. The files are in NetCDF format and contain useful metadata, such as variable long names, so individual variables *can* easily be extracted and evaluated, but researchers should be careful to learn the exact nature and provenance of those variables. Metadata can be misleading.

The files are written at the end of each 6 h hindcast, but contain data with a variety of frequencies, as appropriate for each variable. At the end of each year they are concatenated into year long time series for convenient archiving and later direct use by CESM. They are also compressed for efficient archiving and copying to local computers. Table 4 describes the CESM coupler history files and which CESM components need which ones. An example of a file name is f.e21.FHIST_BGC.f09_025.CAM6assim.011.cpl_0080.**ha2x1d**.2011.nc.gz which can be parsed using Table 3. In addition, the filetypes can be parsed as follows:

h: = (coupler) history file
a,r: = the component which provides the forcing fields (“atm” or “river”)
2: = “to”
x: = all other components
d,h: = the frequency of the data (days or hours). “Day” is actually a 6 h span, due to the length of the hindcasts.
i: = flag that the data is instantaneous, instead of averaged (the default).

NOTE: CESM generally interpolates these data to the start time requested by the user. In order to start a hindcast on the first date of the dataset (2011-01-01-00000), the 2011 forcing files must contain data from the end of 2010. This sometimes raises questions about whether the “time” variable, and “time:units” and “time:bounds” metadata are all correct. They are.

**Table 4:** Which forcing file filetypes are used by which CESM component models in “data atmosphere” mode.

Filetype $\rightarrow$ active component	ha2x1d	ha2x3h	ha2x1h	ha2x1hi	hr2x1d
lnd: CLM, CTSM	Yes	Yes	Yes	Yes	
ocn: POP, MOM	Yes	Yes	Yes		Yes
ice: CICE	Yes	Yes	Yes		
$\text {glc}$^a^: CISM	Yes	Yes	Yes	Yes	
$\text {rof}$^a^: RTM, MOSART	Yes	Yes	Yes	Yes	

^a^Depending on the compset chosen, these components may need forcing files from components in addition to the DATM. In that case, a case can be set up and run to use the DATM forcing files and write out the additional forcing.

### Restart file sets

CESM is able to fully stop and restart with results that are identical to a case where it doesn’t stop. It does this by writing and reading comprehensive “restart” files, which contain a complete description of the state of the model. In the context of ensemble assimilation, it writes a restart file set for each ensemble member. A restart set for one member (INST) for one date is stored in the RDA in a file like f.e21.FHIST_BGC.f09_025.CAM6assim.011.INST.alltypes.YYYY-MM-DD-00000.tar where “alltypes” indicates that a variety of filetypes are contained in it. See the CESM file naming conventions for details about these standard filetypes. The list of files in such a file can be found in Table 5. The variables in some of the files will be described in subsections, below.

**Table 5:** The list of files in a “restart set”, their filetypes, and example file names (CASENAME = f.e21.FHIST_BGC.f09_025.CAM6assim.011). The history files are not required for an exact restart of the model, but are archived with the restart files to maintain continuity of optional time series of (time averaged) fields requested from the case. The relationship between history and restart history files is described in CESM Post Processing Data. The DART inflation restart history files are not used during CESM’s restart process, but are used by the assimilation. They are also archived more frequently with the other DART output as described in “Ensemble mean and spread files”.

Component model	File type	File name
CAM6	Initial	CASENAME.cam_0080.**i**.2020-01-01-00000.nc
CAM6	Restart	CASENAME.cam_0080.**r**.2020-01-01-00000.nc
CAM	Surface restart	CASENAME.cam_0080.**rs**.2020-01-01-00000.nc
CICE	Restart	CASENAME.cice_0080.**r**.2020-01-01-00000.nc
CLM5	Restart	CASENAME.clm2_0080.**r**.2020-01-01-00000.nc
cpl7	Restart	CASENAME.cpl_0080.**r**.2020-01-01-00000.nc
MOSART	Binary restart	CASENAME.mosart_0080.**r**.2020-01-01-00000
DOCN	Binary restart	CASENAME.docn_0080.**rs1**.2020-01-01-00000.bin
CAM	History #0	CASENAME.cam_0080.**h0**.2019-12-31-64800.nc
CLM	History #0	CASENAME.clm2_0080.**h0**.2020-01-01-00000.nc
CLM	History #1	CASENAME.clm2_0080.**h1**.2020-01-01-00000.nc
MOSART	History	CASENAME.mosart_0080.**h0**.2020-00.nc
CLM	Restart history #0	CASENAME.clm2_0080.**rh0**.2020-01-01-00000.nc
CLM	Restart history #1	CASENAME.clm2_0080.**rh1**.2020-01-01-00000.nc
MOSART	Restart history #0	CASENAME.mosart_0080.**rh0**.2020-01-01-00000.nc
DART	Restart history	CASENAME.dart.**rh.cam_forecast_priorinf_mean**.2020-00.nc
DART	Restart history	CASENAME.dart.**rh.cam_forecast_priorinf_sd**.2020-00.nc

These restart sets can be used to start single or ensemble hindcasts, and to start data assimilation experiments. Each ensemble member is an equally likely model state, so they can be chosen at random, unless a specific atmospheric feature is needed, such as the strongest hurricane in the ensemble. They are available every Monday at 00:00 UTC.

#### CAM initial files

In this reanalysis CAM6 is instructed to write out an ensemble of “initial” files at the end of each hindcast. See Table 5 for an example file name. They contain the variables which comprise the DART *prior* state vector, and enough other variables to start a CAM6 hindcast (Table 6). In contrast to the “restart” files, they cannot resume a previous hindcast in a continuous manner. Initial files were chosen over restart files for the storage of the state variables for several reasons:

- smaller size (less than 1/3),
- more intuitive variables (e.g. sensible temperature instead of scaled virtual potential temperature),
- better metadata,
- no need for identical hindcast continuation.

That last reason is because DART changes the model state variables between the end of the previous hindcast and the start of the next, so identical continuation is not possible.

**Table 6:** The CAM6 variables which are written to the initial files. The state variables are those that are directly changed by the assimilation. The rest evolve in response to the changes to the state variables as well as the model physics and dynamics. The “modes” in the MAM4^19^ modal aerosol scheme are “accum”(1), “aitken”(2), “coarse”(3), and “primary_carbon”(4).

Variable name	Description
**CAM6 + DART model state variables**	
PS	Surface pressure
T	Sensible temperature
US	Zonal wind component (on a grid staggered latitudinally 1/2 grid box)
VS	Meridional wind component (on a grid staggered longitudinally 1/2 grid box)
Q	Specific humidity
CLDLIQ	Cloud liquid water (grid box average)
CLDICE	Cloud ice (grid box average)
**Other moisture variables**	
NUMICE	Cloud ice number (grid box average)
NUMLIQ	Cloud liquid number (grid box average)
NUMRAI	Rain number (grid box average)
NUMSNO	Snow number (grid box average)
RAINQM	Rain amount (grid box average)
SNOWQM	Snow amount (grid box average)
**Aerosols**	
DMS	Dimethyl sulfide
H_2_O_2_	H_2_O_2_
H_2_SO_4_	H_2_SO_4_
SO_2_	SO_2_
SOAG	Secondary organic aerosols gas
**MAM4 modal aerosol scheme variables**	
bc_a[1,4]	Black carbon, modes 1 and 4
dst_a[1–3]	Dust, modes 1 through 3
ncl_a[1–3]	Sea salt (NaCl) , modes 1 through 3
num_a[1–4]	Aerosol number density, modes 1 through 4
pom_a[1,4]	Primary-organic aerosols, modes 1 and 4
soa_a[1,2]	Secondary-organic aerosols, modes 1 and 2
so4_a[1–3]	Sulfate (SO_4_ modes 1 through 3

#### Restart files

The initial intended use of these file sets was to provide convenient dates for restarting assimilations due to computing environment difficulties, or to branch experiments, which tested aspects of the assimilation. They will hopefully prove useful for starting (ensemble) hindcast experiments and further CAM assimilation experiments. Due to their low frequency (weekly), and the fact that the contents are not easily or completely described, they do not lend themselves to meaningful evaluation, so no detailed table of variables will be provided here.

For the curious, here is an overview of the file contents. The CAM restart files contain similar variables to the initial files, but many more (184 versus 36). There is no metadata for many of them and some do not even appear in the CAM log file “MASTER LIST”. The CLM restart files have 276 variables, whose metadata includes their long names, and 143 more which lack that. The coupler restart files contain 203 variables, many of which are related to the variables in the coupler history (“forcing”) files. They contain additional fluxes *from* non-atmospheric (or river) components, such as CICE and CLM. The CICE restart files contain 54 variables with no metadata for them. Consult the User’s Guide if needed. The river runoff (MOSART) restart files contain 14 variables on a longitude–latitude grid, with long name metadata.

#### Inflation files

Following Bayes’ theorem, the prior distribution (represented by an ensemble) is combined with the observations at each assimilation cycle to produce a posterior. If the spread of the prior is small due to model and sampling errors or if the observation estimate is overconfident, the posterior will also be overconfident. This is easy to view because after applying Bayes’ rule, the posterior variance will be smaller than both the prior and observation error variances. The easiest way to fix that is to “inflate” the ensemble; increase the spread of the ensemble around its mean, while preserving the mean. In large, complex models such as CAM6, an effective algorithm for doing that is adaptive inflation, which defines an inflation value (and standard deviation) at each model grid point for each state variable, and evolves them in time in order to adapt to the evolving distribution of observations and model state. The inflation can be applied to the prior or posterior ensemble. Studies have shown that prior inflation can mitigate model biases while posterior inflation is effective at reducing sampling errors^20^. Model biases often dominate sampling errors. In this reanalysis study, we use the adaptive prior inflation algorithm described in El Gharamti et al.^14^.

**Figure 5:**
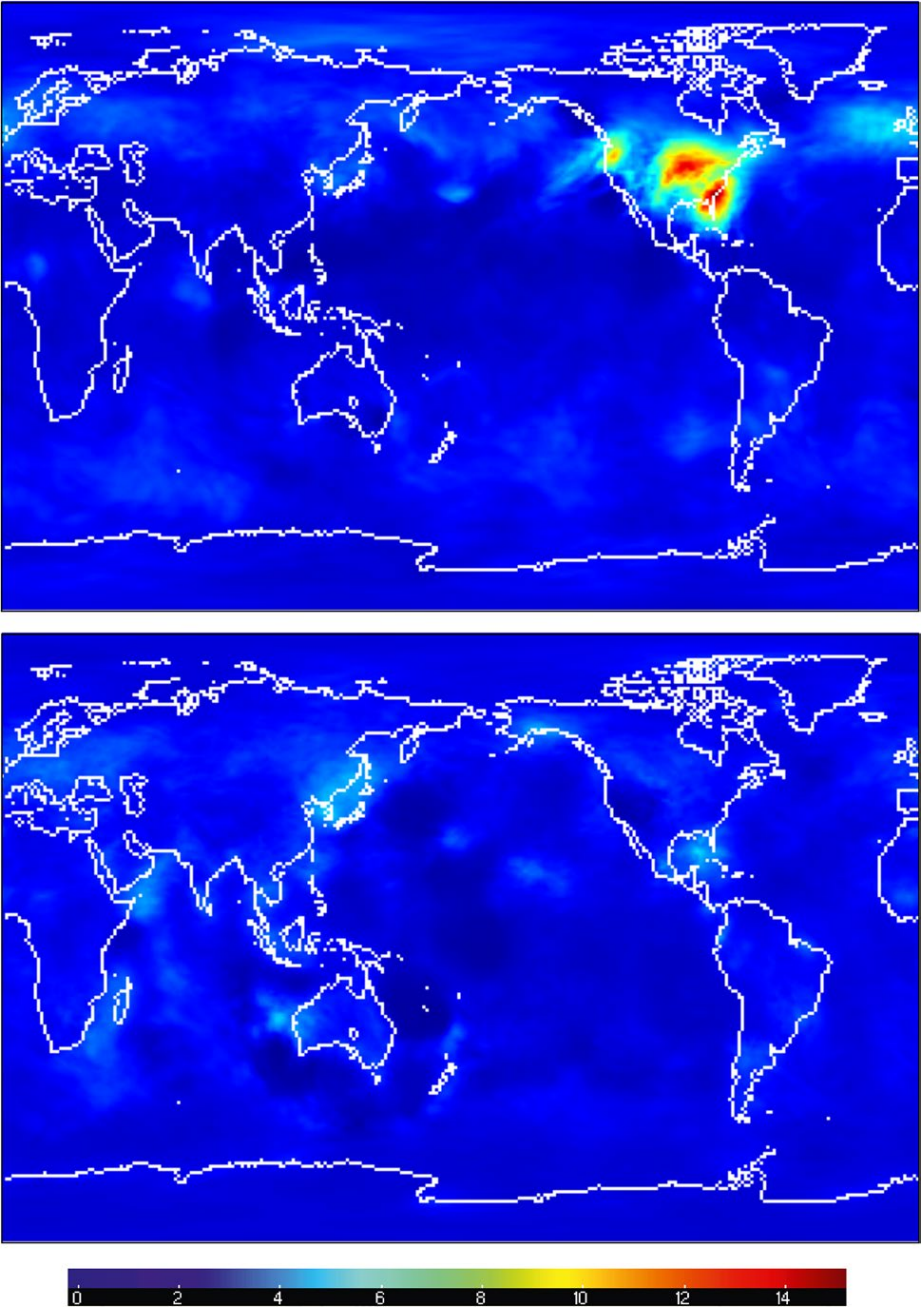
The mean values of the adaptive inflation coefficients for the zonal wind component (US) on the $\approx 200$ hPa (top) and $\approx 860$ hPa (bottom) model levels on Sep. 1, 2016 at 00 UTC. The inflation mean is defined for all model state variables at all assimilation times. It is a dimensionless quantity. Note the relationship between the inflation values at 200 hPa and the density of ACARS temperature observations shown in Fig. 3c. Similarly for the 860 hPa inflation values and the radiosonde US observations in Fig. 3a. The inflation standard deviation is constant (0.6) everywhere for all model state variables, so it is not shown. The figure, including continental outlines, was drawn using ncview (version 2.1.7), which is open source software licensed under the Gnu General Public License version 3.

This evolving inflation data is stored in “inflation restart files” during each assimilation cycle. The product of the algorithm is essentially a distribution, so the inflation is written out as a pair of mean and standard deviation inflation files. This should *not* be interpretted as the inflation of the model mean and the inflation of the model standard deviation; these files describe the state of the inflation algorithm. Examples of those file names are in the restart file Table 5 and have the filetypes rh.cam_forecast_priorinf_mean and rh.cam_forecast_priorinf_sd. These files have variables named the same as the model state variables (see Table 6), but their contents are inflation values, defined by the filetype. The inflation fields are useful for evaluating the assimilation performance, and they are relatively small, so they are archived for every cycle. The subset of these files, which are archived as part of the restart file sets, are in archive files having “infl_log” in their names, e.g. as in the restart files outline. See Fig. 5 for examples of the inflation mean fields.

### Atmosphere model ensembles

The whole ensemble of *prior* model states is archived at least weekly in files of the form f.e21.FHIST_BGC.f09_025.CAM6assim.011.cam_allinst.e.forecast.YYYY-MM-DD-SSSSS.tar (not to be confused with the “alltypes” files in restart sets). The *posterior* versions of the contents of these files are archived in the CAM initial files in the (weekly) restart sets (see Table 5) [NOTE: When the initial files were written they contained the prior model states (see “CAM Initial Files”). After the assimilation they contain the posteriors]. The assimilation innovations can be calculated by subtracting a prior (forecast) variable from its posterior (initial file) version, e.g. for temperature; $T_{innovation} = T_{init} - T_{forecast}$.

NOTE: Similar ensemble files with filetype “preassim” contain the inflated ensemble, which cannot be used to calculate assimilation innovation without deflating the ensemble first, using the inflation values described in “Inflation time series”. This is in contrast to the innovations of the mean, which can be calculated from the ensemble mean of either “forecast” or “preassim”. See “Ensemble mean and spread files”.

### Assimilation files

The program “filter”, which does the assimilation, writes out a variety of files, which were used to evaluate the health of the assimilation, but can also be a source of research data. They are described in detail in the following subsections.

#### Observation sequence files

DART currently requires that the observations to be assimilated and the metadata describing them be packaged into a custom formatted file. The assimilation process generates an ensemble of model estimates of each observation, which are written, with the actual observations, to an “obs_seq_final” file using the same custom format. In this reanalysis the files are binary for compactness.

At the end of each month of assimilation the obs_seq_final files are compressed into a tar archive for archiving in the RDA. The file names have the form f.e21.FHIST_BGC.f09_025.CAM6assim.011.cam_obs_seq_final.YYYY-MM.tar

The observation types, which were assimilated (e.g. RADIOSONDE_TEMPERATURE), are a subset of the types available in the obs_seq_final files. Other types were merely “evaluated” during the assimilation, meaning that filter calculated the ensemble model estimates of the observations, but did not change the model state to fit them better. These are often referred to as “withheld” observations, which provide an independent verification of the assimilation. Table 7 lists the observation types and how they were used.

**Table 7:** The names of the observation types available in the obs_seq_final files can be constructed by combining an entry from the first column (e.g. AIRS_) with an entry from the top row (e.g. TEMPERATURE), e.g. AIRS_TEMPERATURE. That type is the name for retrievals of temperature from the AIRS instrument on the AQUA satellite. QTY is an abbreviation for DART’s “quantity”, which denotes what is being measured, while TYPE denotes the source of the measurement. Each type was either **assim**ilated , **eval**uated , (blank) ignored, or ‘X’ doesn’t exist.

QTY TYPE	TEMPERATURE	U_WIND_ COMPONENT	V_WIND_ COMPONENT	SPECIFIC_ HUMIDITY	ALTIMETER	REFRACTIVITY
ACARS_	assim	assim	assim	X	X	X
AIRCRAFT_	assim	assim	assim	X	X	X
AIRS_	assim	X	X	eval	X	X
GPSRO_	X	X	X	X	X	assim
LAND_SFC_	X	X	X	X	eval	X
MARINE_SFC_					eval	X
RADIOSONDE_	assim	assim	assim	eval	eval	X
SAT_	X	assim	assim	X	X	X

#### Statistical representations of observation space performance

**Figure 6:**
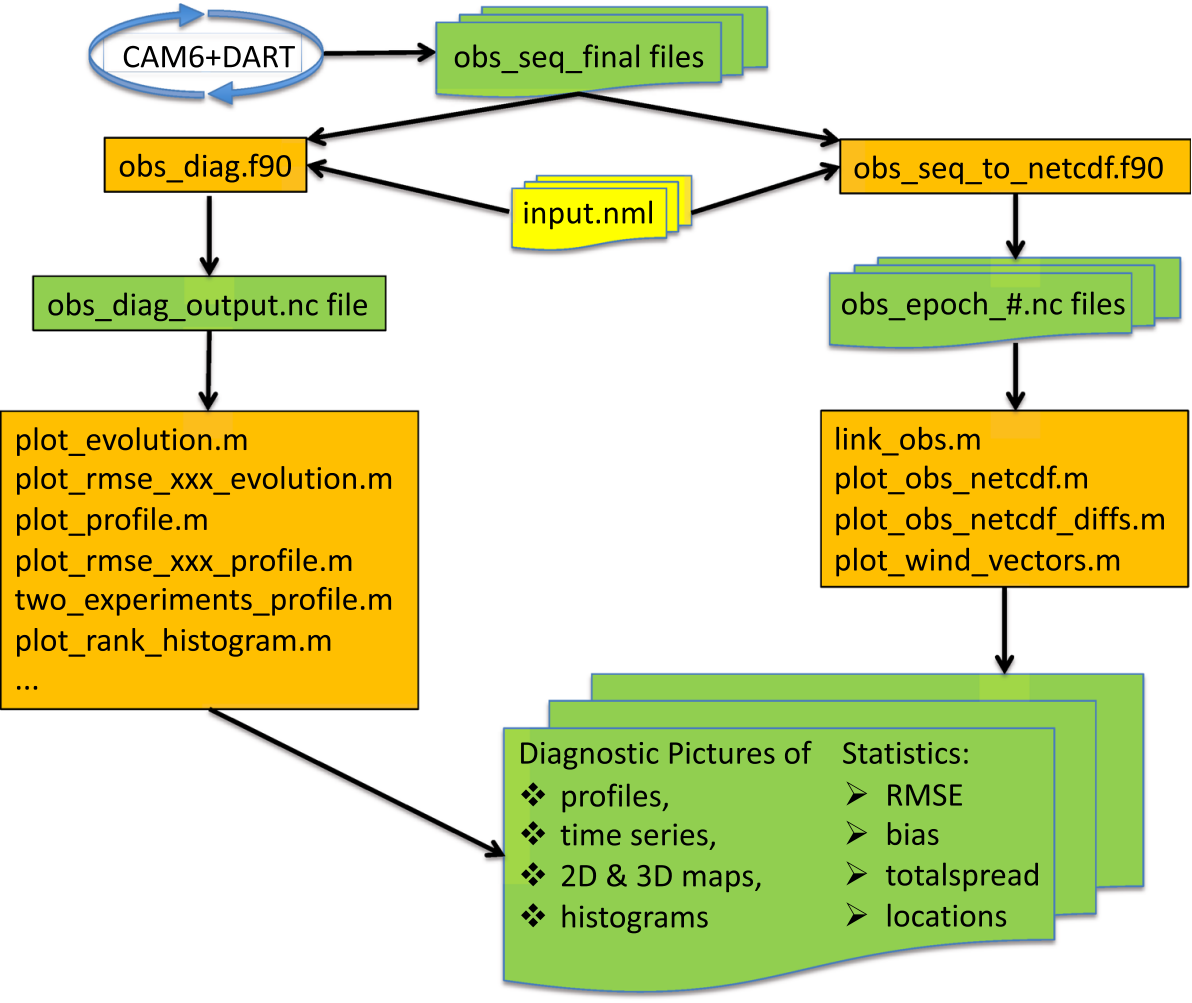
A map of the observation space diagnostics software available in DART. The CAM6+DART cycling (Fig. 2) creates a time series of binary obs_seq_final files, which have a custom format. They are easily converted to NetCDF using DART’s obs_diag.f90 and obs_seq_to_netcdf.f90 programs. The obs_diag_output.nc file has the time series concatenated into it. The obs_epoch_#.nc files map one-to-one to the obs_seq_final files, and are numbered sequentially. Then a wealth of statistics (Table 8) can be calculated and displayed by DART’s suite of Matlab scripts (.m). The resulting .pdf and .ps pictures are suitable for presentations.

In order to calculate the statistics of the observation data in the obs_seq_final files, as discussed in “Observation space diagnostics”, program “obs_diag” harvests data from them, performs some of the statistical analysis, and writes the results into a NetCDF file (“obs_diag_output.nc”). This workflow, and related workflows, are outlined in Fig. 6. Obs_diag enables users to focus the evaluation in many ways. The following features of the evaluation can be specified as inputs to obs_diag:

- observation types
- horizontal and vertical domains
- date range of the data
- statistics or variables displayed (bias, RMSE, total spread, rank histograms, and more)

DART provides Matlab^©^ scripts to read the obs_diag_output.nc files, finish the statistical analysis, and display the results. (see “Assimilation diagnostic files” for guidance about replacing the Matlab^©^ scripts by similar software of the user’s choosing.) The resulting files are packaged into files of the form “Diags_NTrS_YYYY-MM.tgz”, which contain the following files, most of which are described by a template due to the large number of similar names. Not all combinations of TYPE and QTY could be generated; see Table 7 for a map.

- obs_diag_output.nc (statistics generated by program obs_diag)
- script.m (Matlab^©^ script which created the .ps and .pdf files)
- matlab_nc.out (diagnostic output from script.m)
- TYPE_QTY_rmse_bias_evolution_region#.ps
- TYPE_QTY_rmse_totalspread_evolution_region#.ps
- TYPE_QTY_rmse_bias_profile_region#.pdf
- TYPE_QTY_rmse_bias_norm_profile_region#.pdf
- TYPE_QTY_rmse_totalspread_profile_region#.pdf
- TYPE_QTY_rmse_bias_obscount.txt

Where

YYYY: = 2011 $$\ldots $$ 2019
MM: = 01 $$\ldots $$ 12.
TYPE: = ACARS, AIRCRAFT, AIRS, GPSRO, LAND_SFC, MARINE_SFC, RADIOSONDE, SAT
QTY: = TEMPERATURE, U_WIND_COMPONENT, V_WIND_COMPONENT, SPECIFIC_HUMIDITY,
  ALTIMETER, REFRACTIVITY
#: = Regions (defined by latitude in this case) “1”; $$20 N - 90 N $$ , “2”; $$20 S - 20 N $$ , “3”; $$90 S - 20 S $$
NTrS: = the 3 regions: Northern hemisphere, Tropics, Southern hemisphere

**Figure 7:**
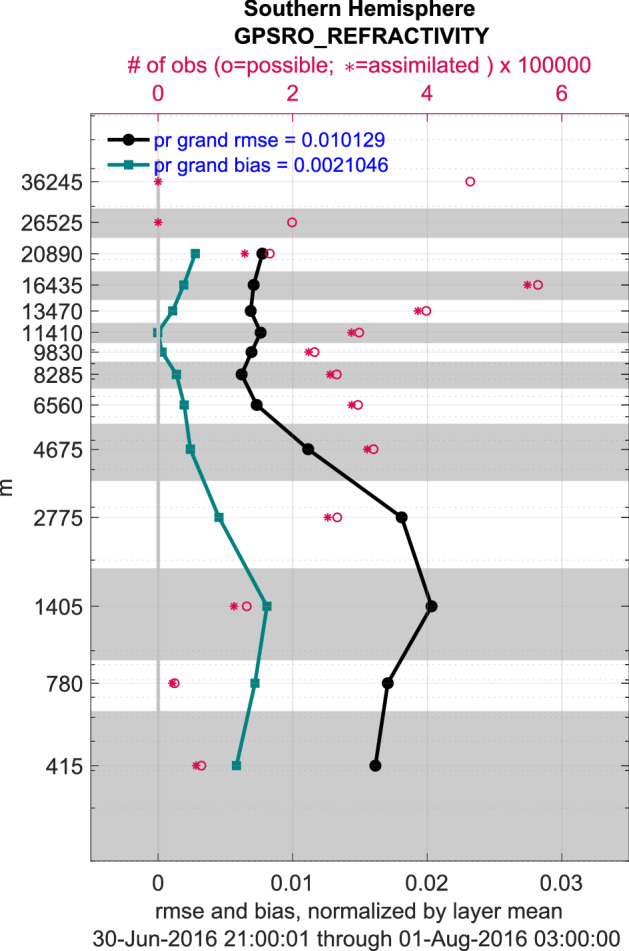
Profiles of the RMSE (black curve) and bias (green curve) of the ensemble mean estimates of the GPS refractivity retrievals (dimensionless) relative to the observed values, which were assimilated in the “Southern Hemisphere” ($20{-}90S$). The profiles have been normalized by the average in each layer. These profiles show the statistics of just the prior (“pr”) model states. The left axis numbers show the height (meters above the surface) of the standard or mandatory pressure levels (assuming a US standard atmosphere). All of the observations in a gray or white band have been binned together for analysis. The “grand” statistics (upper left corner) pool the observations from all layers. The red circles and plusses mark the number of observations available and assimilated in each layer (top axis). The (normalized) RMSE is dimensionless.

An illustration of time series from this type of analysis is in Fig. 4 and is discussed in “Observation space diagnostics”. A more condensed view of some of the data in this reanalysis is shown in Fig. 7. This picture provides examples of many features of the assimilation and evaluation, which are helpful for understanding the data products. The statistics shown there are calculated at the observation locations, so any interpolation is performed on the model state, not on the observations. The vertical dimension is divided into 14 layers, each of which contains a standard or mandatory pressure level of the atmosphere. The layers are not the model vertical levels. The figure also shows the number of observations available and the number assimilated in each layer, which provide guidance about the health of the assimilation (a large fraction assimilated is healthy) and about the trustworthiness of the RMSE and bias (small number assimilated = untrustworthy). Note that there are no observations assimilated in the top two layers, due to a choice to avoid assimilation in the top levels of the model, which are damped and don’t respond well to assimilation.

The observations in Fig. 7 come from low orbit satellites, such as COSMIC, which very accurately measure the transit time of radio signals emitted by Global Positioning System satellites. The times are distorted by the atmosphere, which makes it possible to calculate the atmospheric index of refraction. The index is reported as the observation, but is more accurately called a “retrieval” because it is derived from the raw measurements. Note that, despite the fact that the retrievals are not model state variables, they can be assimilated and provide information which is useful for updating the model state variables. The actual index values are near 1.0, but the reported numbers have the form $n_{rep} = (n_{whole} - 1.0) \times 100$ in order to focus on the variable part of the numbers. The index of refraction is a function of atmospheric density, which decreases exponentially with height. The profiles have been normalized by the average $n_{rep}$ in each layer, so that the curves in the upper layers don’t appear to be “zero”. During this month in this region, all of the biases happen to be $\ge 0$, which reveals that CAM6 has a denser atmosphere than the observations indicate. Further probing reveals that a cold bias explains some of this density bias (not shown). This reanalysis reveals a variety of persistent biases in the model and/or in some of the observation platforms. The RMSE and bias shown in Fig. 7 are two examples of the quantities available in the obs_diag_output.nc files for visualization. The complete list of these “copies” is shown in Table 8, and is taken from the variable CopyMetaData in those files.

**Table 8:** Obs_diag_output.nc files contain variables with a dimension (“copy”), which organizes the statistics of the contents of the observation sequence files (obs_seq_final). They are ordered in that file according to the CopyMetaData variable. Bold text highlights statistics which are displayed in the Diags_NTrS_YYYY-MM.tgz files. Further details can be found in the DART software package; $\ldots$/your_DART/assimilation_code/programs/obs_diag/threed_sphere/obs_diag.html.

Variable	Description	Use(s)
**Nposs**	Number of observations available	Helps assess the assimilation health
**Nused**	Number of observations assimilated	Helps assess the assimilation health
NbigQC	Number of obs rejected due to flag from observation provider	
NbadIZ	Number of excessive innovation Z scores	
NbadUV	Number of unmatched U/V wind pairs	Can reveal problems with the input observation files
NbadLV	Number of obs excluded above the top, below the bottom	Provides context for Nused and Nposs
**rmse**	Root mean square error	Shows the noisiness of the difference of the ensemble mean estimates from the observations. Provides context for other statistics.
**bias**	Average difference of the mean estimates from the observations	Reveals model and/or observation type biases
spread	Combined spreads from ensemble estimates of all observations	Focus on ensembles, or remove from total spread to see observation error
**totalspread**	Combined ensemble spread and observation error of all observations	Provides context for RMSE, bias, and assimilation health
NbadDARTQC	Number of observations not assimilated due to DART criteria	Total observations that failed DART’s quality control checks
observation	Average value of the actual observations in the region + time span	Compare against the ensemble mean
ens_mean	Average value of the ensemble mean estimates of the observations	
N_trusted	Number of observations designated as ’trusted’	These will be assimilated regardless of how different they are from the ensemble estimate.
N_DARTqc_0	Number of observations given DART QC 0	This number were assimilated
N_DARTqc_1	Number given DART QC 1	Only evaluated, not assimilated, following namelist instructions
N_DARTqc_2	Number given DART QC 2	Posterior forward operator failed after prior QC = 1
N_DARTqc_3	Number given DART QC 3	Posterior forward operator failed after prior QC = 4
N_DARTqc_4	Number given DART QC 4	Model estimates of observations could not be calculated
N_DARTqc_5	Number given DART QC 5	DART’s namelists prevented assimilation
N_DARTqc_6	Number given DART QC 6	Prior forward operator failed
N_DARTqc_7	Number given DART QC 7	Observations were too far from the ensemble means
N_DARTqc_8	Number given DART QC 8	DART failed to convert the observations’ vertical coordinates

**Figure 8:**
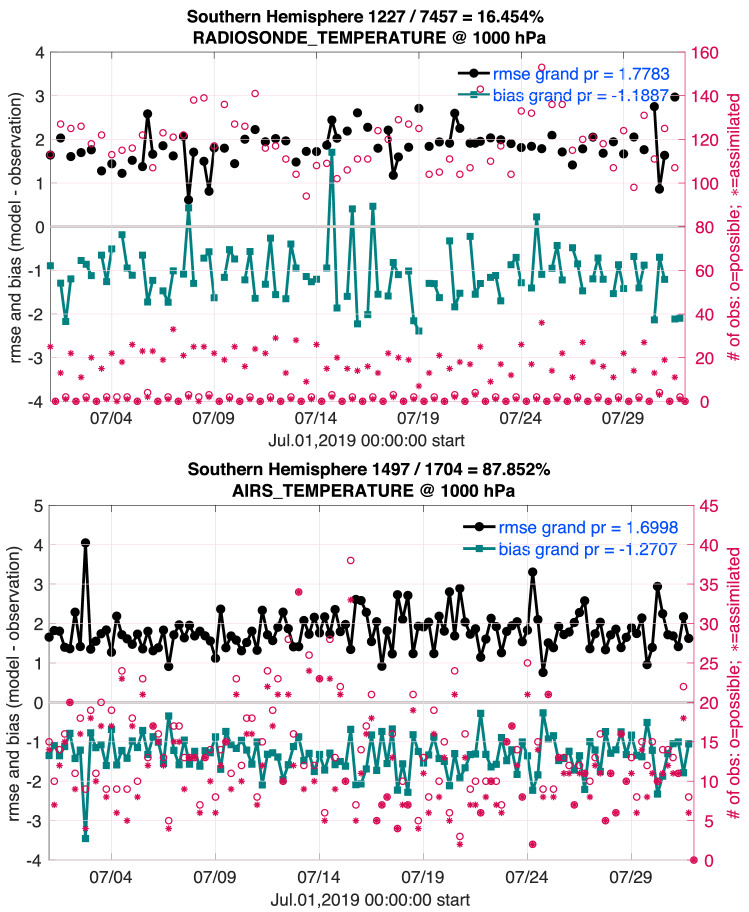
Time series of quantities derived from this reanalysis related to two observation platforms, as discussed in “Statistical representations of observation space performance”. The red circles and asterisks mark the number of observations available and assimilated (right axis) at each time (bottom axis). The black dots and line show the root mean square error of the model ensemble mean estimates of the temperature (K) relative to the observed temperatures (left axis). The green squares and line show the bias (model ensemble mean-observation). The lines in the RADIOSONDE_TEMPERATURE picture are intermittent because times with no data are not plotted. In the region shown there are often no radiosonde observations at 06:00 and 18:00 UTC. These quantities were derived from a subdomain of the model; south of $20^\circ$ S and from the surface to $\sim$ 965 hPa high. The numbers in the legend labeled with “grand” are statistics of the entire month of data. The fraction and percentage in the title represent the fraction of of observations used.

Some of the temperature bias mentioned in the discussion of Fig. 7 can be seen in Fig. 8, which shows pictures from the Diags_NTrS_2019-07.tgz file. In contrast to Fig. 4, these time series show an assimilation that has settled into a stable, healthy pattern. There is no obvious trend in any of the statistics, including the fraction of observations used. The fraction of RADIOSONDE_TEMPERATURE observations used is quite small in the layer displayed here because this reanalysis did not assimilate any observations below the bottom model level, which is ~ 52 m above the surface, and the surface itself is smoothed to be suitable for the model’s 1° resolution. The fraction used is much higher in higher layers and for other observations, such as AIRS (bottom of Fig. 8). A few of the time slots show relatively large (or small) RMSE and bias, which is almost always due to there being very few observations at that time, which can result in randomly bad (or good) agreement with the observations.

Since the bias of the model relative to both observation types is consistently ~ − 1 K, it is fair to conclude that the model probably has a deficiency. This bias persists despite the assimilation frequently pushing the model state towards the observations, so the bias in a free running hindcast would be larger. We should keep in mind the other possibility, that both observation platforms may be biased compared to reality and that the model is closer to the truth than both observation types, but we can’t confirm that from anything in this assimilation. The RMSE represents a combination of the bias and random error. A large portion of this RMSE is due to this bias, implying that the distribution of estimated observations is relatively narrow, which in turn implies that the model estimates are truly inconsistent with the observations and not just “different within the noise”. These biases are of the prior model states, which are in the atmospheric “Surface Forcing Files”. In contrast, the CAM initial files in the “Restart File Sets” contain posterior fields, which will generally have smaller biases because the assimilation has pushed those model states closer to the observations.

#### Ensemble mean and spread files

Filter also calculates the ensemble mean and spread in state space (on the model grid) and writes those to NetCDF files. Those files are written at two “stages”; the “forecast” and “output”, which are the “prior” and “posterior” states of the ensemble, respectively. Those features are summarized in the filetype part of the file name, e.g. “output_sd” means the standard deviation (spread) of the output ensemble. They are available every 6 h. The impact of the assimilation on the model state ensemble mean can be calculated as “innovation = output - forecast”. Note that the “output” files have filetype “.i.” to signal that their contents describe the files which will be used to initialize the next hindcast, while the “forecast” files have filetype “.e.” to signal that they describe model data which will be modified by DART. All of these files contain only quantities describing the model state variables, (see the list in Table 6).

NOTE: Some years have “preassim” files instead of forecast. The ensemble means of those 2 stages are identical, by the design of the inflation algorithm, so both the preassim and forecast means can be used in the mean innovation calculation. This is in contrast to the innovations calculated from ensemble members.

#### Inflation time series

The dart.rh files are concatenations of the inflation restart files, which are described in “Inflation Files” (p11), so they are 6-hourly time series. The rh.cam_**output**_priorinf* filetypes contain the same types of data as the rh.cam_**forecast**_priorinf*, but the fields have been updated by the assimilation. They were used by the subsequent assimilation.

### Land model time series

The CLM namelists instructed it to write out “history” files of fields describing the evolution of plant growth under the influence of an ensemble of realistic atmospheric forcing. The fields were written to two files to separate the time averaging applied to each. Filetype “h0” has instantaneous values. Filetype “h1” has time averaged values. Files were written at the end of each 6 h hindcast, then concatenated into year long time series, and compressed for archiving. The requested fields, along with fields written by default, are listed in Table 9.

**Table 9:** Fields written to CLM5 history files describe the evolution of plant growth. Bold indicates requested fields. The others are written by default. Instantaneous fields are stored in filetype “h0”. Time averaged fields are stored in filetype “h1”.

Variable name	Description
**Instantaneous values**	
**EFLX_LH_TOT**	Total latent heat flux [+ to atm]
**ER**	Total ecosystem respiration, autotrophic + heterotrophic
**HR**	Total heterotrophic respiration
**TSA**	2 m air temperature
ZSOI	Soil depth
DZSOI	Soil thickness
WATSAT	Saturated soil water content (porosity)
SUCSAT	Saturated soil matric potential
BSW	Slope of soil water retention curve
HKSAT	Saturated hydraulic conductivity
ZLAKE	Lake layer node depth
DZLAKE	Lake layer thickness
**Time averaged values**	
**CPHASE**	Crop phenology phase
**GPP**	Gross primary production
**GRAINC_TO_FOOD**	Grain carbon to food
**GSSHALN**	Shaded leaf stomatal conductance at local noon
**GSSUNLN**	Sunlit leaf stomatal conductance at local noon
**NPP**	Net primary production
**NPP_NUPTAKE**	Total carbon used by nitrogen uptake in FUN
**PLANT_NDEMAND**	Nitrogen flux required to support initial GPP
**QVEGT**	Canopy transpiration
**TLAI**	Total projected leaf area index

### Files that force CAM

The component set chosen for this reanalysis uses a “data ocean” as a time evolving boundary condition which forces CAM6. While that dataset completely specifies the influence of the oceans on CAM, other datasets supply partial influences. These are added to related, internally generated fields, which are read from the initial files. One example is aerosols, which evolve according to model physics and chemistry, but also have contributions from sources outside of the atmosphere, such as industrial emissions. The files which provide this “external forcing” are provided in a CESM archive. Installation of CESM on most supercomputers includes at least parts of this archive in a location which is accessible to everyone. If these files are not in that archive, request that they be downloaded from the CESM data repository, which can be accessed by following instructions in the CESM User’s Guide. The namelist variables which specify the needed files are describe in the CESM2 CAM6 namelist variables page.

#### Sea surface temperature and ice coverage

The data which CESM reads to provide SSTs and sea ice coverage (but not thickness) was taken from the RDA dataset (ds277.7) before 2020, when it was replaced by files which have data valid at 12:00 UTC instead of 0:00 UTC. The 0:00 UTC data used in this reanalysis are archived in the RDA ds345.0^18^ dataset avhrr-only-v2.2011-202001_0Z_filled_c200810.nc.gz.

#### Aerosols and green house gases

For this CESM component set and for years before 2015 CESM chooses default files which contain historical data. For hindcasts in 2015 and later, there is a choice of using data in a “CYCLICAL” mode, which uses a specified year of data, or using CMIP6 scenario projections and the “INTERP_MISSING_MONTHS” mode. The scenarios are very similar during the first few years after 2014, so the choice is not of central importance. We chose files containing data from scenario “SSP3-7.0”, which specifies a moderate increase in green house gas. The non-default files used in this reanalysis are listed in Table 10.

**Table 10:** Information about files which CAM6 reads to get data describing external contributions to chemical and aerosol constituents. SERIAL; extract data which has the current model date. CYCLICAL; extract data from the specified year from the file. INTERP_MISSING_MONTHS; extract data from the 2 years in the file, which bound the current model year, and from the current model month of those years. For example, if the curent model date is 2018-06-12 and the file has years 2015 and 2020 in it, then data from 2015-06 and 2020-06 will be used to interpolate to 2018-06. The 6 digits following the _c encode the creation date (YYMMDD). The other 6 digit numbers are year + month combinations.

Namelist variable	Value	[File contents]
srf_emis_type	CYCLICAL	Surface emitted aerosols added to those in the CAM initial file
srf_emis_cycle_yr	2014	
srf_emis_specifier	22 files with “surface_1750-2015_0.9x1.25” in their names.	
prescribed_ozone_type	CYCLICAL	$\mathrm {O_3}$ concentration
prescribed_ozone_cycle_yr	2014	
prescribed_ozone_file	ozone_strataero_WACCM_L70_zm5day_18500101-20150103_CMIP6ensAvg_c180923.nc	
prescribed_strataero_type	CYCLICAL	MAM4 mode concentrations: dust(3), NaCl(3), $\mathrm {SO_4(1-3)}$
prescribed_strataero_cycle_yr	2014	
prescribed_strataero_file	ozone_strataero_WACCM_L70_zm5day_18500101-20150103_CMIP6ensAvg_c180923.nc	
flbc_type	SERIAL	Lower boundary fluxes of $\mathrm {CO_2 , CH_4 , N_2O , CFC11 , CFC12}$
flbc_file	LBC_2014-2500_CMIP6_SSP370_0p5degLat_GlobAnnAvg_c190301.nc	
tracer_cnst_type	INTERP_MISSING_MONTHS	Chemical concentrations of $\mathrm {O_3, OH, NO_3, HO_2}$
tracer_cnst_file	tracer_cnst_halons_3D_L70_2014-2101_CMIP6-SSP5-8.5_c190307.nc	
ext_frc_type	INTERP_MISSING_MONTHS	
ext_frc_specifier H2O	H2OemissionCH4oxidationx2_3D_L70_1849-2101_CMIP6ensAvg_SSP3-7.0_c190403.nc	
ext_frc_specifier num_a1	emissions-cmip6-ScenarioMIP_IAMC-AIM-ssp370-1-1_num_so4_a1_anthro-ene_vertical_mol_175001-210101_0.9x1.25_c20190222.nc	
ext_frc_specifier so4_a1	emissions-cmip6-ScenarioMIP_IAMC-AIM-ssp370-1-1_so4_a1_anthro-ene_vertical_mol_175001-210101_0.9x1.25_c20190222.nc	

#### Land model

The CLM5 component of this reanalysis employed two external forcing files. Population density was read from firedata/clmforc.Li_2018_SSP3_CMIP6_hdm_0.5x0.5_AVHRR_simyr1850-2100_c181205.nc in SERIAL mode. Land use was read in CYCLICAL mode (year 2015 data) from file landuse.timeseries_0.9x1.25_SSP5-8.5_78pfts_CMIP6_simyr1850-2100_c181209.nc.

### Log files

Further details about the hindcast model are in the weekly “infl_log” files. These include log files from the overall job (“cesm”), one of the 80 couplers (“cpl_0001”), and one each of the 5 nonstub components; cpl_0001, ice_0001, lnd_0001, ocn_0001, rof_0001. Log files from the assimilations are not archived in the RDA collection, but are available on NCAR’s Campaign Storage file system for a limited time. The most important aspects of the assimilation setup and results are described here and in the RDA dataset.

## Technical validation

The intent of this project is to provide a useful representation of Earth’s actual weather for use in Earth system research, especially ensemble data assimilation. It is not to provide the best atmospheric reanalyses possible. As in any reanalysis, the fidelity to the weather depends on the types and numbers of observations at the time and in the area of interest, as well as on the ability of the model to simulate the atmospheric state. The validity of the data is described by some of the dataset itself, as noted in the “Validation” section. DART also provides methods for further evaluating these data products when a more focused evaluation is needed. See “Observation space diagnostics”, “Statistical representations of observation space performance”, and “Ensemble mean and spread files” for examples and discussion. The suitability of this data for an application should be carefully evaluated independently of the evaluations available in this dataset. There is likely enough diagnostic data in this dataset that you can compare these results to reanalyses produced by operational centers.

### Monthly observation space evaluation

Much of the technical evaluation of this reanalysis is contained in the monthly, observation space pictures generated by DART’s Matlab^©^ scripts. That process is described in “Methods” and “Statistical representations of observation space performance”.

**Figure 9:**
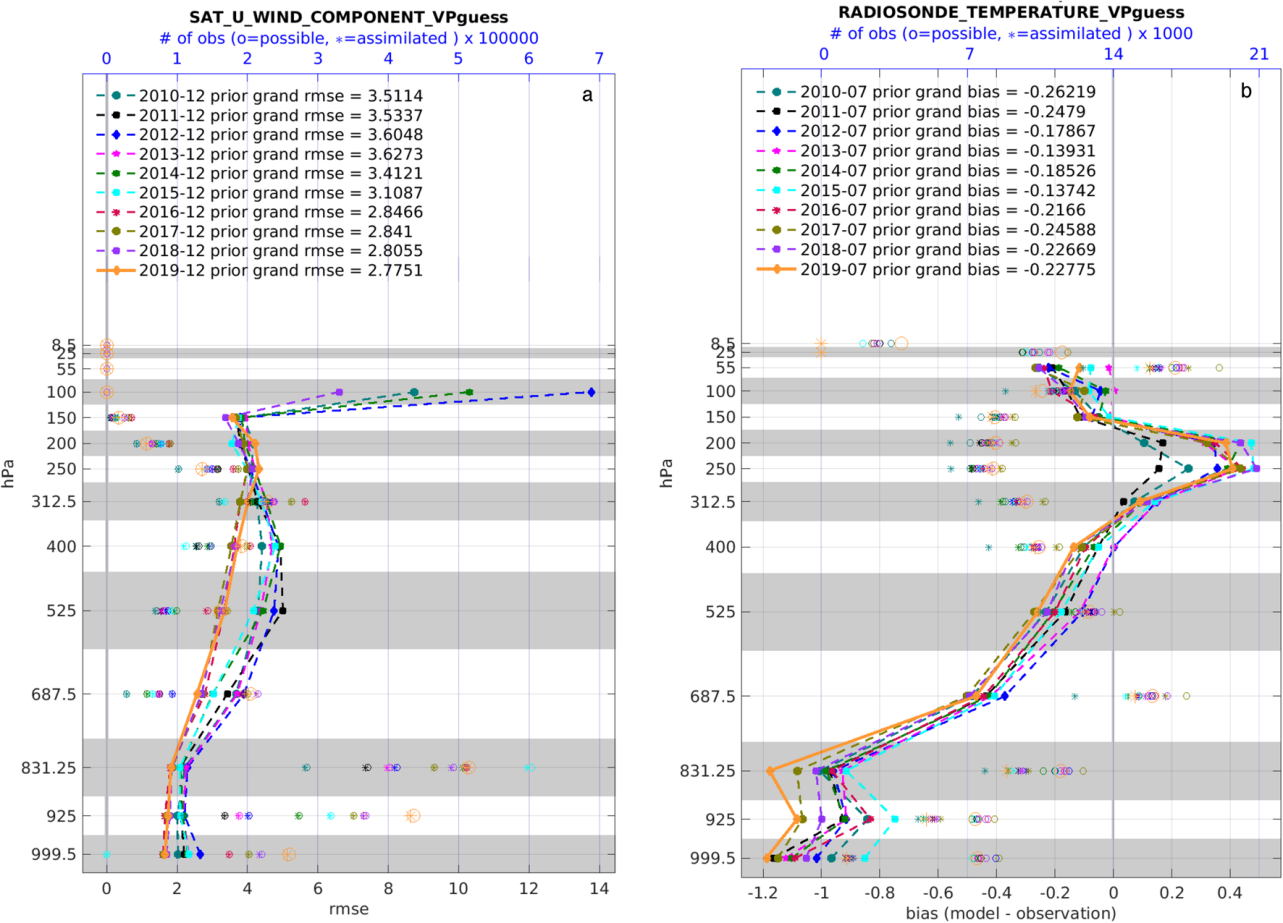
(**a**) The vertical profiles of the RMSE ($\text{ms}^{-1}$) of the zonal wind component of the prior model ensemble relative to the zonal, satellite, cloud drift, wind observations in December of each of 9 years (plus the spin-up year of 2010). It also shows the number of observations available ($\circ$) and used ($*$) during each December. Colors of all symbols are labeled in the legend. The “grand rmse” number is the RMSE relative to all of the observations in the month (i.e. all levels combined). (**b**) same as ‘(**a**)’, but for the bias relative to radiosonde temperature observations ($\text {K}^\circ$) in July .

In addition to examining individual months, we compared the same month from all years to evaluate long term trends in the assimilation quality. Examples are shown in Fig. 9, which are not included in the reanalysis archive (e.g. Diags_NTrS_YYYY-MM.tgz) but could be recreated from the available data. Note that in Fig. 9a there is a dramatic decrease in the RMSE in layers 525 hPa and 400 hPa during the last 4 years and an increase in the number of observations assimilated. We believe that that this is due to an improvement in 2016 in the algorithm used to calculate the wind from the movement of clouds. The RMSE values in the 100 hPa layer should be ignored because there were hardly any observations there, so the statistics are not robust.

Figure 9b shows generally larger temperature biases relative to radiosonde observations in the lower layers in recent years. We have not found an explanation for this trend, but have not seen any evidence that the reanalysis is failing. This sort of behavior could be the subject of further research.

### Ensemble spread

**Figure 10:**
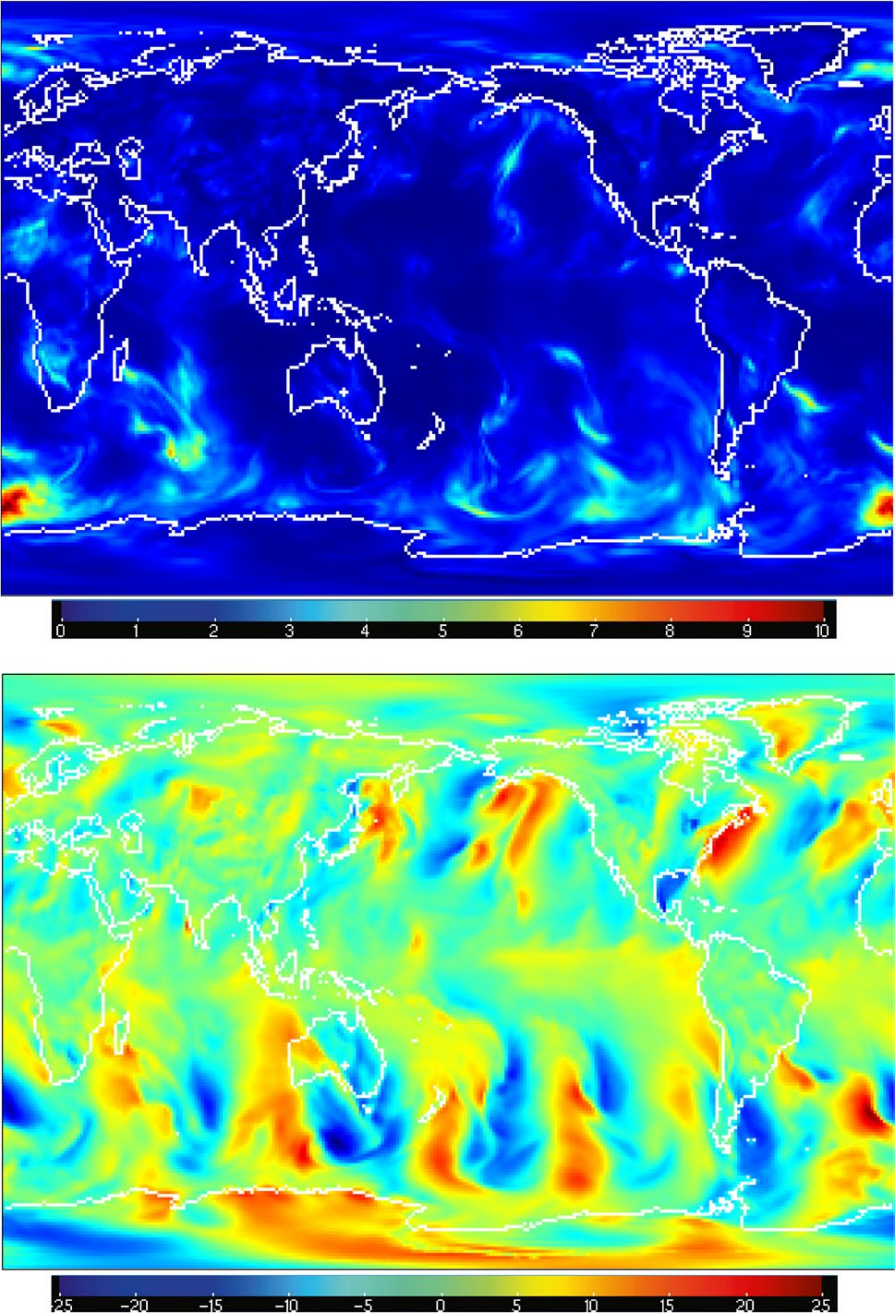
Top; the posterior ensemble standard deviation (“spread”) of the meridional wind component (VS) on 2019-11-01-00000 at the model’s lowest level (8 hPa above the surface). The data range from $\sim 0.034$ to $\sim 10.6\, \text{ms}^{-1}$. Bottom; the same, but for the ensemble mean. The data range from $\sim -23.6$ to $\sim 23.8\, \text{ms}^{-1}$. The figure, including continental outlines, was drawn using ncview (version 2.1.7), which is open source software licensed under the Gnu General Public License version 3.

The observation space diagnostics discussed in the previous sections show the fidelity of the model state ensemble mean to the observations. They basically answer the question “Is the model’s most likely estimate of the weather consistent with the observations?” An equally important question is “Is the consistency due to chance or to high certainty in the model ensemble?” That question is answered by the prior ensemble spread, which varies widely with location, time, and the atmospheric field of interest. An example is shown in Fig. 10. There is some correlation between the weather (ensemble mean, bottom figure) and the ensemble spread (top figure) in areas which are not well observed, such as the southern oceans. Well observed areas, such as over continents and down wind from them, have very small ensemble spread, indicating high confidence in the means.

### Use of the surface forcing files in CESM

Multiple tests have demonstrated that the coupler history files (“forcing files”) can be read by CESM and provide atmospheric forcing for data atmosphere configurations of CESM (“DATM”), such as compsets whose short names start with “I” (CLM), “C” or “G” (POP2), and “D” or “G” (CICE). See Table 4 from “Surface Forcing Files”, Fig. 1, and the CESM component set web page for details.

### Reproducibility

A reanalysis such as this is a complex activity, involving many layers of software, evolving computing environments, and human intervention. Fortunately, the core activity is comparison of model output against observations, so the end product is closely tied to reality and is verifiable. Our intent in providing this dataset to the community is to prevent the needless reproduction of such a large amount of data. However, some reproducibility exercises are valuable for verification of the data products, the software used to create them, and how well data users understand it all. There are at least two varieties of reproducibility that are desirable from the various subsets of data; bitwise or statistical. Although the hindcast model and DART are designed to be capable of bitwise reproducibility, the fundamental nature and goal of ensemble data assimilation is a statistical description of the physical system. Two assimilations which use fundamentally the same hindcast model and the same assimilation algorithm, but different initial ensembles, should generate ensembles which are statistically indistinguishable after the typical spinup period. Conclusions drawn from such ensembles are robust and meaningful, while conclusions based on individual members or details of the differences between the the ensembles are generally not robust and are suspect.

#### Bitwise

The extent to which the results are bitwise reproducible will depend on many factors, including availability of the same or equivalent hardware, compilers, and libraries. The farther removed in time the reproduction effort is, the less likely it is to succeed, and the more effort it will take. For example, at one point some desirable output was lost during the archiving process. We attempted to recreate the data, after a significant computer upgrade, by recompiling the programs with the updated compilers to ensure consistency with the updated environment. We were unable to bitwise reproduce the data in the available time, but we discovered that the original executables, compiled *before* the upgrade, still reproduced earlier results exactly.

#### Statistical

When attempting to reproduce this reanalysis it is necessary to define an acceptable disagreement between the reproduction and the original. There is a range of standards which could be used, depending on the intended use. Some examples are, in roughly increasing precision:

- Are the differences between the means smaller than the model state changes between assimilation times?
- ...smaller than the combined uncertainties of the two distributions?
- ...smaller than the assimilation innovations?
- Are the distributions indistinguishable using formal statistical methods of comparison, which take into account the possibility that the distributions are not random draws from the continuous distribution which represents the atmospheric variable?

The last, strictest standard is difficult to define and calculate, even in the case of ensemble hindcasts with no data assimilation, as described by Milroy et al.^21^. We are not aware of any adaptations of those techniques to ensemble data assimilation.

## Usage notes

### Surface forcing files

CESM uses the coupler history (DATM surface forcing) files by reading their names and locations from “stream” files in the CASEROOT directory where the experiment is set up. Their names have the form “user_datm.streams.txt.prescribed_INST” where INST is an “instance number” (ensemble member, padded with 0s). In the context of a DART assimilation experiment, these stream files are created during the set up of the CASE from template files found in:

${DART}/models/{cice,clm,POP}/shell_scripts/streams.txt

In those directories there are also scripts with “setup” in their names, which can be used to set up assimilation experiments, including the creation of the user_docn.streams.txt.prescribed_INST files. Before use, the coupler history files (e.g. f.e21.FHIST_BGC.f09_025.CAM6assim.011.cpl_0001.ha2x3h.2011.nc.gz) will need to be decompressed using ‘gunzip‘.

### Restart file sets

The restart file sets can be used to start a simple hindcast by decompressing one of them into an accessible “REFDIR” directory and setting CESM’s env_run.xml:

CONTINUE_RUN = FALSE

RUN_TYPE = hybrid

RUN_REFCASE = f.e21.FHIST_BGC.f09_025.CAM6assim.011

RUN_REFDIR = where the restart file set has been decompressed

RUN_REFDATE = the YYYY-MM-DD chosen as the date of the initial condition data. This can be a different year and day from the desired start date, but should be in the same month.

RUN_REFTOD = 00000

The restart file sets can be used to start an atmospheric *assimilation* by decompressing an *ensemble* of them into an accessible “REFDIR” directory and following the usual DART set up procedures, as outlined in the DART tutorials and scripts, such as ${DART}/models/cam-fv/shell_scripts/cesm2_1/*setup*. This is not a trivial exercise, and will be done much more efficiently by becoming familiar with DART before attempting it.

### Assimilation diagnostic files

If additional observation space diagnostics are needed, they can be derived from the f.e21.FHIST_BGC.f09_025.CAM6assim.011.cam_obs_seq_final.YYYY-MM.tgz files using scripts and programs in DART, as outlined in Fig. 6. Some of those are described in “Observation sequence files”. In particular, program obs_diag can be rerun using different horizontal regions, time spans, vertical layers, etc. These are controlled by fortran namelist parameters in input.nml:obs_diag_nml.

DART has numerous Matlab^©^ (.m) scripts to further process and display the contents of the NetCDF files produced by obs_diag.f90 and obs_seq_to_netcdf.f90. The latter creates files whose contents can be viewed as multiple linked frames with data selection capabilities. Several scripts display three-dimensional, rotatable renderings of the data. See DART’s Diagnostics page for illustrations and details.

Once the NetCDF files are created, software packages other than Matlab^©^ could be used. In that case the Matlab^©^ scripts can serve as helpful templates for dealing with the many issues which must be resolved to create useful pictures. Users have begun contributing Python code for the manipulation of DART diagnostic output. It is not as advanced as DART’s Matlab^©^ environment, but we expect it to develop rapidly. Please contact us for the current status (dart@ucar.edu).

### Land model time series

These NetCDF files can be evaluated and manipulated by the usual tools. The “h0” file variable time series are defined on the CAM lon-lat grid, so they can be easily displayed. The “h1” file variable time series have the CLM “pft” dimension (plant functional type), which is too large for display ($\approx 800{,}000$) without subsetting. There are many pfts in each grid box, distributed among the land surface types^22^.

### Use cases

Several uses of this dataset have been described in previous sections:

$$\triangleright $$
Use of the Surface Forcing Files in CESM (see Fig. 1 for an illustration).
$$\triangleright $$
Restart File Sets describes initial conditions for hindcasts.
$$\triangleright $$
assimilation innovations can be calculated from the data described in “Atmosphere model ensembles” and “Ensemble mean and spread files”
$$\triangleright $$
Assimilation Files describes how to see and generate statistics describing the assimilation.
$$\triangleright $$
Land Model Time Series describes the plant growth variables in the CLM5 history files (Table 9)
$$\triangleright $$
Comparison of these reanalyses with others, but see comments in “Technical Validation”, or combination with them, as in the Multi-Reanalysis Ensemble v2.

This large dataset has a unique combination of an 80 member ensemble, 1° global resolution, 6-hourly frequency, and 9 year time span, which provides opportunities for robust statistical analysis and use as a machine learning training and verification dataset. The data differs from model generated training sets in that it is constrained to represent the atmosphere (and plant growth characteristics), rather than the model formulation.

The observation space diagnostics can be used to help identify biases in observation platforms. The dataset already contains biases of the reanalysis relative to a variety of observation types, as a function of season, time of day, height, and broad latitude regions. Further refinement of the influences on the biases can be generated, as needed.

### Code availability

DART software is managed in github.com/NCAR/DART, where users can access the tag which was used to generate this reanalysis; “DS345.0”. The general web site for the version of CESM used here is http://www.cesm.ucar.edu/models/cesm2. The specific version can be found in github.com/kdraeder/cesm; the cesm2_1_forcing_rean branch. That branch includes a SourceMods tar file, which needs to be unpacked before building CAM6. There are minor changes to the CESM scripting to make it run more efficiently in ensemble mode. These are available in github.com/kdraeder/cime; in the cime_reanalysis_2019 branch. This cime needs to be imported into the CESM source code directory during the package build process, as described in the CESM documentation. Some of the csh scripts use NCO NetCDF operators, which are freely available. DART’s Matlab scripts are compatible with version R2018b, Update 5 (9.5.0.1178774), 64-bit (maci64), license number 473564.
